# The Development of the Military Service Identification Tool: Identifying Military Veterans in a Clinical Research Database Using Natural Language Processing and Machine Learning

**DOI:** 10.2196/15852

**Published:** 2020-05-25

**Authors:** Daniel Leightley, David Pernet, Sumithra Velupillai, Robert J Stewart, Katharine M Mark, Elena Opie, Dominic Murphy, Nicola T Fear, Sharon A M Stevelink

**Affiliations:** 1 King's Centre for Military Health Research King's College London London United Kingdom; 2 Institute of Psychiatry, Psychology and Neuroscience King's College London London United Kingdom; 3 South London and Maudsley NHS Foundation Trust London United Kingdom; 4 Combat Stress Letherhead United Kingdom; 5 Academic Department of Military Mental Health King's College London London United Kingdom; 6 Department of Psychological Medicine Institute of Psychiatry, Psychology and Neuroscience King's College London London United Kingdom

**Keywords:** natural language processing, machine learning, military personnel, electronic health care records, mental health, veteran

## Abstract

**Background:**

Electronic health care records (EHRs) are a rich source of health-related information, with potential for secondary research use. In the United Kingdom, there is no national marker for identifying those who have previously served in the Armed Forces, making analysis of the health and well-being of veterans using EHRs difficult.

**Objective:**

This study aimed to develop a tool to identify veterans from free-text clinical documents recorded in a psychiatric EHR database.

**Methods:**

Veterans were manually identified using the South London and Maudsley (SLaM) Biomedical Research Centre Clinical Record Interactive Search—a database holding secondary mental health care electronic records for the SLaM National Health Service Foundation Trust. An iterative approach was taken; first, a structured query language (SQL) method was developed, which was then refined using natural language processing and machine learning to create the Military Service Identification Tool (MSIT) to identify if a patient was a civilian or veteran. Performance, defined as correct classification of veterans compared with incorrect classification, was measured using positive predictive value, negative predictive value, sensitivity, F1 score, and accuracy (otherwise termed Youden Index).

**Results:**

A gold standard dataset of 6672 free-text clinical documents was manually annotated by human coders. Of these documents, 66.00% (4470/6672) were then used to train the SQL and MSIT approaches and 34.00% (2202/6672) were used for testing the approaches. To develop the MSIT, an iterative 2-stage approach was undertaken. In the first stage, an SQL method was developed to identify veterans using a keyword rule–based approach. This approach obtained an accuracy of 0.93 in correctly predicting civilians and veterans, a positive predictive value of 0.81, a sensitivity of 0.75, and a negative predictive value of 0.95. This method informed the second stage, which was the development of the MSIT using machine learning, which, when tested, obtained an accuracy of 0.97, a positive predictive value of 0.90, a sensitivity of 0.91, and a negative predictive value of 0.98.

**Conclusions:**

The MSIT has the potential to be used in identifying veterans in the United Kingdom from free-text clinical documents, providing new and unique insights into the health and well-being of this population and their use of mental health care services.

## Introduction

### Veterans

Estimates of the United Kingdom’s military veteran population, defined by the British Government as those who have served in the military for at least one day [1], are approximately 2.5 million, equivalent to approximately 5% of household residents aged 16 years or older in the United Kingdom [2]. UK military veterans receive health care provision from the National Health Service (NHS) alongside civilians, with care recorded in local, regional, and national electronic health care records (EHRs) [3]. EHRs—structured and unstructured (ie, free text)—can be used to evaluate disease prevalence and surveillance, to perform epidemiological analyses and investigate the quality of care, and to improve clinical decision making [4,5].

Veterans of the United Kingdom experience a range of mental health problems (estimates range from 7% to 22% across psychiatric conditions), some resulting from their experiences in the line of duty [6]. A large UK cohort study set up to investigate the health of serving personnel and veterans has also shown that veterans report higher levels of probable posttraumatic stress disorder and alcohol misuse than serving personnel [7]. Recent research suggests that 93% of veterans who report having a mental health difficulty seek some form of help for their problems, including informal support through family and friends [8]. However, there is no national marker in the UK EHRs to identify veterans, nor is there a requirement for health care professionals to record it, making it difficult to evaluate the unique health care needs of those who have served in the UK Armed Forces [9]. Furthermore, the ability to identify veterans would allow for comparisons between civilian and military cohorts and for direct comparison of their physical and mental health.

In England and Wales, only two studies exist, which analyze secondary care delivered through the NHS for Armed Forces personnel. In the first study, Leightley et al [3] developed a method to link the EHRs of military personnel in England, Scotland, and Wales (3 nations of the United Kingdom). This study used a longitudinal cohort consisting of serving personnel and veterans to establish a link to national EHRs (England, Scotland, and Wales). Then, statistical analyses were performed to identify the most common reasons for admission into hospital, diagnoses, and treatment pathways. The second study, by Mark et al [10], on which this study is based, systematically searched for veterans using a military-related search term strategy on free-text clinical documents using a manual approach. Although this approach could identify veterans, it was time consuming as searches were performed manually. Each of these studies highlighted a need for novel methodological development for the identification of veterans, with natural language processing (NLP) and machine learning showing great promise [11-13]. This would enable the automatic identification of veterans without the need for manual annotation and validation.

### Neutral Language Processing

NLP approaches cover wide-ranging solutions to the analysis of text, such as retrieval, analysis, transformation, and classification of text, such as those found in EHRs and free-text clinical documents [13,14]. NLP subthemes, such as text mining, are represented as a set of programmatic rules or machine learning algorithms (eg, automated learning from labeled data) to extract meaning from naturally occurring text (eg, human-generated text) [11,14]. The result is often an output that can be interpreted by humans and that can be processed computationally more efficiently [15]. It may be possible to apply NLP for the identification of veterans, if not already defined from structured fields, such as a flag for denoting veteran status, for which, in the United Kingdom, are rarely coded [10]. The ability to identify veterans at scale could significantly improve our understanding of their health and well-being and navigation of care pathways and allow for the exploration of the long-term impacts of service.

### This Study

NLP tools have been used extensively in military health research, predominantly in the United States, for the detection of veteran homelessness and clinical diagnosis [16-19]. However, to the best of our knowledge, no tools exist to identify veteran status using either a rule-based or machine learning approach. The aim of this study was to describe the development of the Military Service Identification Tool (MSIT) for the identification of veterans using free-text clinical documents and to evaluate the tool’s performance against a manually annotated dataset (gold standard). This study was inspired by the study by Fernandes et al [14], but we proposed a different approach to the way in which features are generated and used for training machine learning classifiers and the annotation of the training and testing data and the way in which we evaluate the performance of MSIT across different classifiers.

## Methods

### Data Source—Clinical Record Interactive Search System

The Clinical Record Interactive Search (CRIS) system provides deidentified EHRs from the South London and Maudsley (SLaM) NHS Foundation Trust, a secondary and tertiary mental health care provider serving a geographical catchment of approximately 1.3 million residents of 4 south London boroughs (Lambeth, Southwark, Lewisham, and Croydon) [20]. The CRIS system has supported a range of research projects [20-23]. Many of these have aimed to answer specific clinical or epidemiological research questions and have drawn on particular subpopulations being identified in the database, such as ethnic minorities and those with Alzheimer disease [24,25].

Ethical approval for the use of CRIS as an anonymized database for secondary analysis was granted by the Oxford Research Ethics Committee (reference: 08/H0606/71+5). This study has been approved by the CRIS Patient Data Oversight Committee of the National Institute of Health Research (NIHR) Biomedical Research Centre (reference: 16-056).

The documents used in this study are Correspondence, which are created by clinical staff to provide a summary of admission or care received and are sent to a patient’s general practitioner and, in some cases, to the patient themselves. Correspondence were used as they routinely provided a detailed history of a patient’s life events including employment history.

### Study Design

There are approximately 300,000 correspondence documents available in CRIS. Owing to the large volumes of data, a subset was extracted for the development of the MSIT. This subset (hereafter termed personal history dataset) was extracted using the personal history detection tool, which has been developed by the CRIS team [26]. This tool identifies documents that have a subheading or section entitled personal history (or similar) before extracting the proceeding text (see Textbox 1 for an example). Each personal history record contains an outline of each patient’s life events since birth (eg, educational attainment, childhood adversity, employment, and relationship information). Each record is written by a clinician. The personal history dataset contains 98,395 documents sampled from records recorded in CRIS since 2006, which was the first year the CRIS database was operational.

After an informal scoping exercise, discussions with NLP experts with experience of using CRIS and timing constraints of the study, the decision was made to retain only 6672 documents (hereafter termed gold standard dataset), which represented 4200 patients (civilian: 3331 and veteran: 869). A patient could have multiple documents that represent different time points of care. The decision to retain 4200 patients (which in total had 6672 documents) was made considering resource limitations of the study, which included staff time to annotation and balancing patient privacy as to only process a minimum number of records to allow us to archive the study aim. A sample size calculation was not performed because of these considerations.

For evaluating the performance of MSIT, a decision was made to retain 66.00% (4470/6672 documents) of the dataset for training, and the remainder 34.00% (2202/6672 documents) was used for testing and evaluation. Patients were sampled to either the training or testing; dataset a patient’s documents would not appear in both samples. There is no defined approach for determining the size of the training and testing sets needed, with most research using ad hoc reasoning depending on data, financial, time, or personal constraints [27]. This study followed an iterative approach to the development of the MSIT, first by developing a structured query language (SQL) rule–based method, with lessoned learned, such as which keywords cause misclassification, informing the development of MSIT.

**Synthetically generated personal history statement by the research team for a female patient whose father and husband served in the military. X denotes personal identifier being removed. Owing to patient confidentiality, we were not able to share real examples from the personal history dataset.**

Mrs X was born in X. Her father was a Normandy D-Day veteran who had sustained a bullet wound to his left arm during the war. He subsequently worked as a bus driver in and around X. Mrs X describes her upbringing as old-fashioned, traditional and one of poverty. She describes her school years as happy and fun and says she got on well with her parents. She acknowledged that during her teenage years that she was difficult to manage. She met her husband X while on holiday in X; X was stationed there in a military unit conducting NATO exercises. After they began a relationship, in 1983, they moved to X. Mrs X worked in various jobs including in a supermarket and as a hotel receptionist, before taking an administrative job in academia.

### Generating the Gold Standard Dataset and Interrater Agreement

A set of classification rules for the annotation of each document was developed and agreed upon by DL, EO, DP, and SS. The Extensible Human Oracle Suite of Tools (University of Utah) software package was used to perform annotations [28]. The following words and phrases were annotated: (1) those that described a patient’s military service (ie, “he served in the Army”), (2) those that described an individual other than the patient’s military service (ie, “dad served in the Forces”), and (3) those that may cause confusion (ie, “Navy Blue”). This led to the creation of a gold standard dataset, which contained veterans- and civilians-annotated free-text clinical documents. Veterans were labeled as such based on a clear statement that the patients themselves had served in the military. The protocol, including classification rules, is available on request from the corresponding author.

### Developing a Rule-Based Approach for Veteran Identification

Civilians and veterans were classified using the SQL rule–based method based on a corpus of known words and phrases related to military service (see Multimedia Appendix 1). The corpus was composed of (1) primary search terms: common words or phrases used to describe military service, (2) secondary search terms: used to validate that the document describes a patient who has served in the military, and (3) exclusion terms: used to exclude documents that may describe another person’s military service and not the patient’s military service.

The SQL rule–based method was developed using a combination of the research team’s expert knowledge of the military, relevant research literature, and analysis of personal history statements. The gold standard training dataset was used to refine the SQL rule–based approach. The code was iteratively tested on the training set, reviewed, and refined to ensure full coverage of known military words and phrases. The SQL rule–based method operated by searching for the occurrence of a primary search term in a document. If the term was found, text surrounding the term would be extracted (up to 50 characters, where available). The extracted text was then evaluated against a list of secondary terms to classify the document as a civilian document or a veteran document. The SQL rule–based approach informed the development of the MSIT.

### Developing the Military Service Identification Tool

A machine learning classification framework was used to create MSIT. It was developed in Python using the Natural Language Processing Toolkit (version 3.2.5) [29] and Scikit-learn (version 0.20.3) [30]. The gold standard dataset was preprocessed to remove (1) punctuations (using regular expressions), (2) words/phrases related to another individual’s military service (these were required to exactly match those in the gold standard annotated dataset), (3) stop words and frequently occurring terms (except military terms), and (4) word/phrases that may cause confusion with correctly identifying a veteran. The remaining features were then converted into term frequency-inverse document frequency (tf-idf) features.

The classification framework was trained to identify veterans based on the use of military terms and phrases with the outcome being binary (1: veteran and 0: not a veteran). A training set of 4470 annotated documents was used to select a machine learning classifier. There is sparse literature on which machine learning algorithms are best suited for specific tasks, not only in the field of NLP but also in areas such as health care, agriculture, and security [31-34]. To ensure the appropriate selection of the classifier used for the MSIT, a comparison was made based on 10-fold cross-validation accuracy using tf-idf features as an input of the following machine learning classifiers (which are part of the Scikit-learn package): random forest, decision tree, linear support vector classifier, support vector classifier, multinomial Naïve Bayes, k-nearest neighbor, logistic regression, and multilayered perception. Each machine learning classifier used default parameters. Linear support vector classifier obtained the highest accuracy (see Table 1; accuracy=0.95; SD 0.01; 95% CI 0.94-0.95) and was used as the machine learning classifier for MSIT.

To improve the true positive rate of the MSIT and to reduce the potential for false positives, a postprocessing of the linear support vector classifier outcome was applied based on the SQL rule–based approach described earlier, as has been used in similar studies [14]. For each document that was predicted as being that of a veteran, an SQL operation was performed to ensure the document used a military term of phrase (eg, “joined the army,” “left the army,” and “demobbed from the army”).

**Table 1:** Machine learning classifier n-fold cross-validation accuracy, SD, and 95% CI based on the gold standard training dataset of 4470 documents.

Classifier	Accuracy	SD	95% CI
Random forest	0.84	0.01	0.83-0.84
Decision tree	0.91	0.03	0.89-0.92
Linear support vector classifier	0.95	0.01	0.94-0.95
Support vector classifier	0.84	0.01	0.83-0.84
Multinomial Naïve Bayes	0.90	0.02	0.88-0.91
k-nearest neighbor	0.89	0.02	0.87-0.90
Logistic regression	0.88	0.04	0.85-0.90
Multilayered perception	0.94	0.02	0.92-0.95

### Availability of Materials and Data

The datasets used in this study are based on patient data, which are not publicly available. Although the data are pseudonymized, that is, personal details of the patient are removed, the data still contain information that could be used to identify a patient. Access to these data requires a formal application to the CRIS Patient Data Oversight Committee of the NIHR Biomedical Research Centre. On request and after suitable arrangements are put in place, the data and modeling employed in this study can be viewed within the secure system firewall. The corresponding author can provide more information about the process.

A Jupyter Notebook demonstrating the tool with artificial data can be found in the link provided [35].

### Statistical Analyses

All analyses were performed using Python version 3.5 with standard mathematical packages and Scikit-learn (version 0.20.3) [30]. Cohen kappa values are presented for civilian and veteran annotations separately, with a two-tailed statistical test applied to determine the significance of the finding. Machine learning classifier 10-fold cross-validation was reported as the highest accuracy obtained, with SD and 95% CI reported to represent the n-fold result. Document characteristics were reported as the average frequency in which words, sentences, whitespaces, stop words, and nonalphanumeric across documents were stratified by civilian and veteran. The most frequent military terms and phrases annotated during the study were restricted to the top 5 and reported as a count with percentage out of the denominator. For evaluating the SQL rule–based approach, the algorithm was tested by measuring the output results against the results from manual annotations (the gold standard testing dataset), allowing for computation of positive predictive value, negative predictive value, sensitivity, F1 score, and accuracy at a document level. For evaluating MSIT, each classifier model was tested by measuring its results against the results from manual annotations (the gold standard testing dataset), allowing for computation of positive predictive value, negative predictive value, sensitivity, F1 score, and accuracy at a document level.

In this study, positive predictive value was defined as the proportion of correctly identified true veterans over the total number of true veterans identified by the classifier. Negative predictive value was defined as the proportion of correctly identified true civilians over the total number of true civilians identified by the classifier. Sensitivity was defined as the proportion of true veterans identified by the classifier over the total number of actual veterans (identified by manual annotation). F1 score considers both positive predictive value and sensitivity and produces a harmonic mean, where the best value lies at 1 and the worst value lies at 0. Accuracy was measured using the Youden Index, which considers sensitivity and specificity (summation minus 1), which results in a value that lies between 0 (absence of accuracy) and 1 (perfect accuracy).

## Results

### Annotation

**Figure 1:**
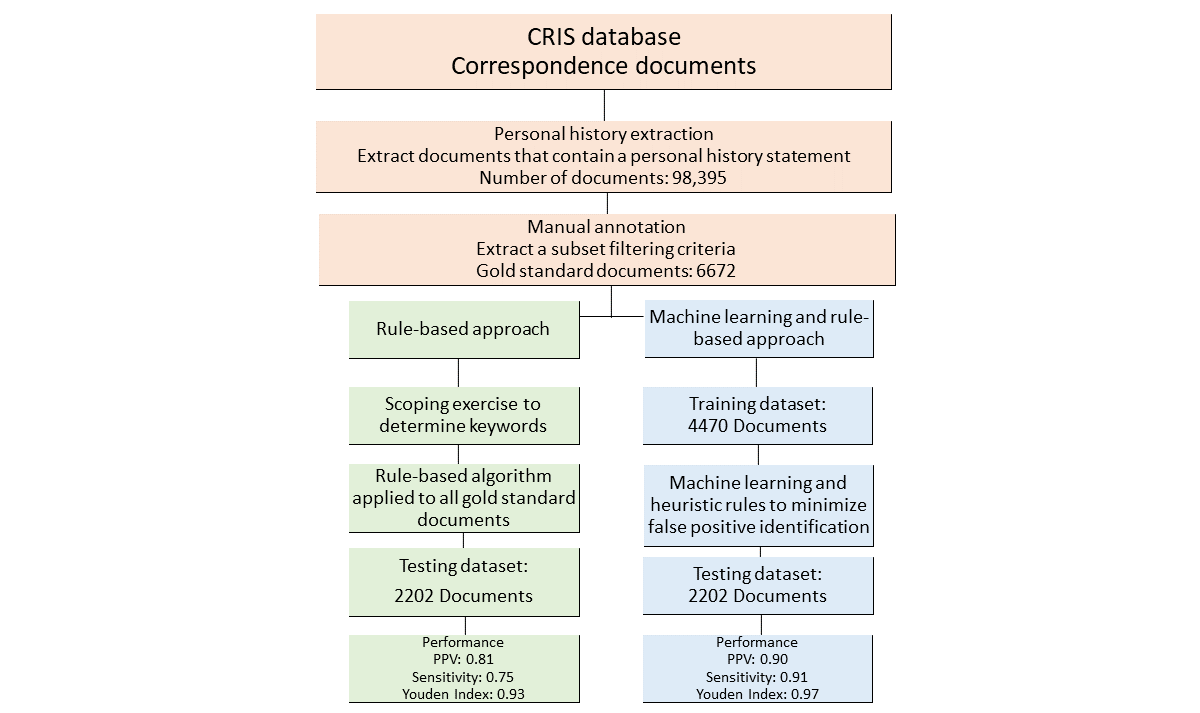
Flow diagram of the Military Service Identification Tool. Correspondences are used to define any communications between a patient and clinical staff or between clinical staff members. CRIS: Clinical Record Interactive Search; PPV: Positive Predictive Value.

An iterative approach to developing MSIT was employed. See Figure 1 for a flow diagram of the MSIT and evaluation process. The datasets used in this study were independently annotated by DL, EO, and a researcher (see Acknowledgments section), with acceptable interrater agreement as indicated by a Cohen kappa of 0.83 for veterans and 0.89 for civilians (*P*=.15).

### Document Characteristics

Of the 6672 documents annotated to generate the gold standard dataset, there were 5630 civilian and 1042 veteran documents. Descriptive characteristics (see Table 2) indicate that often civilian documents had more words, sentences, stop words, and nonalphanumeric characters.

A total of 2611 words and 2016 phrases that describe a patient’s military service were annotated (see Tables 3 and 4). Most of the words and phrases annotated described the service branch (eg, “served in the army,” “national service in the RAF,” “demobbed from the army,” and “was a pilot in the RAF”), with only a small number including the length of service (eg, “served for two years in the army,” “served two years for national service,” and “demobbed from the army after two years”).

**Table 2:** Document characteristics including frequency and mean (SD) for annotated personal history statements stratified by civilian and veteran status.

Characteristic	Civilian (n=5630), mean (SD)	Veteran (n=1042), mean (SD)
Words	223.76 (152.30)	197.20 (114.63)
Sentences	13.80 (8.91)	12.40 (6.50)
Whitespaces	237.99 (162.77)	208.38 (119.65)
Stop words	32.04 (11.45)	30.09 (9.92)
Nonalphanumeric characters	26.59 (20.14)	22.22 (14.28)

**Table 3:** Top 5 occurring military words identified during manual annotation of the gold standard training dataset.

Military words (n=2611)	Value, n (%)
“Army”	553 (21.18)
“National Service”	445 (17.04)
“RAF”	225 (8.62)
“Navy”	166 (6.36)
“Veteran”	104 (4.00)

**Table 4:** Top 5 occurring military phrases identified during manual annotation of the gold standard training dataset.

Military phrases (n=2016)	Value, n (%)
“Joined the army”	167 (8.28)
“Left the army”	122 (6.05)
“Demobbed from the army”	101 (5.00)
“National service in the army”	65 (3.22)
“Two years in the army”	64 (3.22)

### Performance: Positive Predictive Value, Sensitivity, and Accuracy

The performance of each approach was evaluated against the manually annotated gold standard test dataset producing positive predictive value, negative predictive value, sensitivity, F1 score, and accuracy statistics. The gold standard test dataset contained 2202 documents, which included 1882 civilian and 320 veteran documents (see Tables 5 and 6).

The SQL rule–based approach correctly identified 262 veteran documents, incorrectly identified 87 civilian documents as veteran documents, and incorrectly identified 58 veteran documents as civilian documents. Misclassification was because of the rigidity of the keywords used to search the records, with confusion observed between the individual’s serving status and a family member’s status. For example, phrases such as “had served” were used to describe another person’s military service, such as father or brother. This resulted in an overall accuracy of 0.93, a positive predictive value of 0.81, a negative predictive value score of 0.95, a sensitivity of 0.75, and an F1 score of 0.78.

During the initial development of the MSIT, model sensitivity was skewed toward commonly occurring words. To overcome this bias, a 4-step preprocessing step was introduced to identify and remove these frequent words and phrases, punctuation, and stop words, which improved positive predictive value and sensitivity of the tool (training dataset: positive predictive value=0.78 and sensitivity=0.88). To further improve the prediction of the tool and reduce the potential for false positives, a postprocessing step was introduced to ensure a military word or phrase was present in the documents predicted as describing a veteran. The addition of this step improved positive predictive value and sensitivity of the MSIT (training dataset: positive predictive value=0.82 and sensitivity=0.91).

Applying MSIT to the gold standard test dataset correctly identified 290 veteran documents, incorrectly identified 30 civilian documents as veteran documents, and incorrectly identified 27 civilian documents as being a veteran document. Misclassification was observed, with manual inspection of the documents revealing that the military-related terms were used to describe events, occupations, or items for civilians such as “Legion” or “Mess Hall.” This created confusion with the classifier. This resulted in an overall accuracy of 0.97, a positive predictive value of 0.90, a negative predictive value of 0.95, a sensitivity of 0.91, and an F1 score of 0.91. Additional analyses were conducted using the leave-one-out methodology (see Multimedia Appendix 1).

**Table 5:** Confusion matrix indicating the performance of the structured query language rule-based approach and the Military Service Identification Tool (MSIT). The MSIT includes pre- and postprocessing.

Label	Structured query language rule–based approach		Military Service Identification Tool	
	Veteran	Civilian	Veteran	Civilian
Veteran	262	58	290	30
Civilian	87	1795	27	1855

**Table 6:** Structured query language–based approach and Military Service Identification Tool (MSIT) performance result comparison for detecting veterans using the gold standard test dataset. The MSIT includes pre- and postprocessing.

Performance metric	Structured query language rule–based approach	Military Service Identification Tool
Positive predictive value	0.81	0.90
Negative predictive value	0.95	0.98
Sensitivity	0.75	0.91
F1 score	0.78	0.91
Youden Index	0.93	0.97

## Discussion

### Principal Findings

This research has demonstrated that it is possible to identify veterans from free-text clinical documents using NLP. A tool to identify veterans and civilians is described, which performed well, as indicated by high positive predictive value, sensitivity, and accuracy results. To the authors’ knowledge, this is the only study to have developed, applied, and tested NLP for the identification of veterans in the United Kingdom using a large psychiatric database. The MSIT presented superior results to the SQL rule–based approach developed because of the former’s ability to adapt to different military terms. The SQL rule–based approach was, on the other hand, fixed on set keywords.

This is the first study that seeks to identify military veterans from a case register in the United Kingdom using NLP and machine learning. Although military literature is sparse, NLP techniques have been used in the detection of sexual trauma, in the detection of temporal expressions in medical narratives, and for screening homelessness [16,17,19]. Although it is difficult to compare our study with the aforementioned studies, similar methodologies are employed. This includes each developing a gold standard manually annotated dataset, developing a set of rules to support identification, and finally generating features from free text. Although this study used linear support vector classification, as it was determined to be the most optimal, Reeves et al [16] used a maximum entropy classifier to detect temporal expressions. Outside of the military literature, Fernandes et al [14] sought to identify suicidal attempts using a psychiatric database with support vector machines; they were able to detect suicidal attempts with a sensitivity of 0.98, which is higher than what was achieved in this study (MSIT: 0.91). Other studies have compared different classification algorithms for clinical NLP tasks with varying conclusions—achieving optimal performance is highly task-dependent and use-case–dependent [36,37].

The ability to identify veterans could provide insights into the physical and mental health of military personnel and their navigation through, and use of, health care services, including primary and secondary services. This would overcome the current need to either manually identify veterans or to perform large-scale cohort and data linkage studies, such as that by Leightley et al [3]. EHR-based case registers, such as CRIS, function as single, complete, and integrated electronic versions of traditional paper health records [3]. These registers have been positioned as a new generation for health research and are now mandatory in the United Kingdom [3]. The methodological advantages of case registers—including their longitudinal nature, largely structured fields, and detailed coverage of defined populations—make them an ideal research and surveillance tool [38]. EHRs in mental health care provide extremely rich material, and analysis of their data can reveal patterns in health care provisions, patient profiles, and mental and physical health problems [3,39]. EHRs are advantageous for investigating vulnerable subgroups within the wider population [20-22], potential for developing digital interventions [40] and to support data-driven decision making [11].

### Strengths and Limitations

An important strength of this work was the exploitation of NLP, which is advantageous for automating the process of identification and reducing the possibility of human error and bias. Considering the focus of this study, this is the first time that NLP has successfully been used to identify veterans from free-text clinical documents using detailed occupational history that clinicals routinely record. The MSIT described in this work does not rely on any codes (clinical or otherwise) or structured fields, which broadens its application to others, such as diagnosis and occupation detection. Furthermore, veterans may not always be willing or think it is necessary to state their veteran status, particularly in the United Kingdom, which has no department for veterans’ affairs. As such, NLP is advantageous, as it may pick up veterans based on small details that are discussed and recorded during clinical interactions rather than having to rely on disclose of veteran status by an individual upon registration with clinical services.

It must be noted that there are several limitations to the tool described in this work. First, the study relied on patients’ self-reporting that they have served in the military, which could be influenced by the patient’s mental health or failing memory. Second, the need for a clinician to ask a patient’s military status and for this to be accurately recorded in the patient notes. Third, the accuracy of recording by the clinician could have had a negative impact on MSIT’s performance or could result in misidentification of veterans. Fourth, the MSIT relied on the personal history section being present in a correspondence, which may limit scalability. Fifth, although different approaches to stating veteran service were annotated, spelling and additional permutations were not considered. This could limit the generalizability of the algorithms on other datasets. Sixth, identified veterans were not validated against the Ministry of Defence databases or contacted directly to validate veteran status. Seventh, a sample size calculation was not computed for this study. This was because of resource limitations; as a result, this could limit the generalizability of the algorithms on other datasets. Finally, documents were misclassified, often because of military vernacular being used by civilians and/or the clinician or because a family member had served in the military and not the patient. Further work should be undertaken to improve reliability and reduce the rate of misclassification.

### Conclusions

We have shown that it is possible to identify veterans using either an SQL-based or NLP- and machine learning–based approach. Both approaches are robust in correctly identifying civilians and veterans, with high accuracy, sensitivity, and negative predictive values observed. The MSIT has the potential to be used in identifying veterans in the United Kingdom from free-text clinical documents, providing new and unique insights into the health and well-being of this population and their use of mental health care services. Despite our success in this work, the tools are tailored to the CRIS dataset, and future work is needed to develop a more agnostic framework.

## Appendix

Multimedia Appendix 1 Supplementary material.

## Abbreviations

CRIS: Clinical Record Interactive Search
EHR: electronic health care record
MSIT: Military Service Identification Tool
NHS: National Health Service
NIHR: National Institute of Health Research
NLP: natural language processing
SLaM: South London and Maudsley
SQL: structured query language
tf-idf: term frequency-inverse document frequency
