# Supplementary material for: The Development of the Military Service Identification Tool: Identifying Military Veterans in a Clinical Research Database Using Natural Language Processing and Machine Learning
Source: JMIR Med Inform. 2020 May 25;8(5):e15852. doi: 10.2196/15852 (PMC7281146; doi:10.2196/15852)
Supplement: Multimedia Appendix 1 [file medinform_v8i5e15852_app1.pdf]

Supplementary Material

Table 1: Primary search terms used for the development of the tools.

**Search term**	**Comments**
join*	
enlist*	
conscript*	
demob*	
discharge*	Mainly used in context “discharged from ward”, etc
serv*	eg served, service
mobil*	Mainly occurs as “mobility”. One observed use: “mobilised for military service”
army	
armed force*	
airforce	“air force” or “airforce”
marine*	
military	
navy	
naval	
RAF	“RAF”, “R A F”, “R.A.F.” or “R. A. F.”
RN	“RN”, “R N”, “R.N.” or “R. N.”
TA	
ATS	“ATS” or “Auxiliary Territorial Service”
WRAF	
WAAF	
WREN*	WRNS. Exclude “wrench”
WRNS	
WRAC	
H4H	“H4H” or “Help for Heroes”
combat stress	
squadron	
veteran	

Table 2: Secondary search terms used for the development of tools.

Include	Exclude	Exclude (family member)
British **Army**	Salvation **Army**	father
Territorial **Army**	Church **Army**	mother
	Land **Army**	parent
Royal **Navy**	British **Army** Schools	brother
Royal **Marines**		sister
Royal **Air Force**	**army** fatigues	uncle
		aunt
active **service**	Merchant **Navy**	dad
army **service**	Mercantile **Marine**	
ex-**service**		husband
**veteran**	**military** boarding school	partner
military **service**		spouse
national **service**	**join**er*	
naval **service**	**joint***	
**service**man	**wrench**	
**service**woman	ob**served**	
submarine **service**	re**served**	
veterans **service**	de**served**	
	**served** & prison	
	**served** & custodial	
	Iranian **Air Force**	
	US **Army**	
	US **Marine** Corps	
	Sri Lankan **army**	
	Sri Lankain **army**	
	Polish **military**	
	Idi Amin	
	refugee	

Table 3:Additional edge-case terms used for the development of the rule-based tool to identify veterans.

Search term	Comments
squad	RAF **squadron** (leader) x5<br>death squad x1
soldier	19/33 related to military service
sailor	2/6 related to military service
airman	7/8 **not** related to military service (eg chairman, repairman)
active duty	2/2 related to military service
infantry	16/16 related to military service
posted	27/36 related to military service
deploy*	10/14 related to military service
peace keeping	No records found
Afghan*	8/21 relate to military service
Iraq	11/20 relate to military service
Falklands	6/7 relate to military service
Northern Ireland	30/50 related to military service
Bosnia	9/9 related to military service
Kosovo	1/5 related to military service
Korea	5/10 related to military service
Gulf War	5/7 related to military service
WW2	5/11 related to military service
WWII	10/21 related to military service
World War	81 records returned
called up	28/28 related to military service
fought in	4/4 related to military service
served in	80/81 related to military service (exclude “observed in”)
Regiments	Royal Artillery<br>Royal Engineers<br>Women’s Royal Army Corps<br>Royal Fusiliers<br>Royal Army Service Corps<br>Royal Green Jackets<br>Royal Military Police<br>Army Education Corps<br>Royal Army Pay Corps<br>Royal Electrical and Mechanical Engineers<br><br>Exceptions<br>Royal Bank of Scotland<br>Royal Bethlem Hospital<br>“Joined the Royal Army for short period”?

Table 4: Aggregated results for leave-one-out validation methodology applied to the final machine learning classifier reported with 95% Confidence Interval.

	**Military Service Identification Tool**	
	**Veteran**	**Civilian**
**Veteran**	803	48
**Civilian**	62	3287
**Positive predictive value**	0.94 (CI: 0.91-0.95)	
**Negative predictive value**	0.98 (CI: 0.92-0.98)	
**Sensitivity**	0.92 (CI: 0.89-0.93)	
**F1 score**	0.93 (CI: 0.91-0.93)	
**Youden Index**	0.97 (CI: 0.94-0.98)	
